# Development of a Combined Micro-Macro Mechanics Analytical Approach to Design Shape Memory Alloy Spring-Based Actuators and Its Experimental Validation

**DOI:** 10.3390/s21165506

**Published:** 2021-08-16

**Authors:** Aniello Riccio, Carmine Napolitano, Andrea Sellitto, Valerio Acanfora, Mauro Zarrelli

**Affiliations:** 1 Department of Engineering, University of Campania “Luigi Vanvitelli”, 81031 Aversa, CE, Italy; aniello.riccio@unicampania.it (A.R.); carmine.napolitano@studenti.unicampania.it (C.N.); valerio.acanfora@unicampania.it (V.A.); 2 National Research Council of Italy (CNR), Institute for Polymers, Composites and Biomaterials (IPCB), 80055 Portici, NA, Italy; mauro.zarrelli@cnr.it

**Keywords:** shape memory alloy, analytical models, SMA actuators, experimental analysis

## Abstract

In this work, an analytical procedure for the preliminary design of shape memory alloy spring-based actuators is investigated. Two static analytical models are considered and interconnected in the frame of the proposed procedure. The first model, based on the works from An, is able to determine the material properties of the SMA components by means of experimental test data and is able to size the SMA component based on the requirements of the system. The second model, based on a work from Spaggiari, helps to design and size an antagonist spring system that allows one to obtain the geometric characteristics of springs (SMA and bias) and the mechanical characteristics of the entire actuator. The combined use of these models allows one to define and size a complex SMA actuator based on the actuation load requirements. To validate the design procedure, static experimental tests have been performed with the entire SMA actuator.

## 1. Introduction

The use of shape memory alloys (SMAs) as smart materials is continuously increasing due to their great application potential, in particular, in engineering fields related to civil structures, mechanical assemblies, and aerospace components [1,2,3]. Moreover, additional applications can be found in robotics as SMAs easily fulfill the requirement of smaller and lighter components [4,5].

The most valuable capability of a shape memory alloy is to recover an initial configuration after a deformation has occurred by applying a temperature variation. This effect can be exploited to generate a useful stroke for a mechanical actuator. Indeed, SMAs have been widely investigated and have been used in many applications, such as the actuation of rigid joints, the fabrication of micropumps, moving robotic arms, moving wing trailing edges, the actuation of separation devices for satellites, and the actuation of odontology devices [6,7,8,9].

In general, to efficiently use SMA components, it is mandatory to understand the issues related to the manufacturing and properties variation of shape memory alloys [10]. The intrinsic capability of shape memory alloys them recover an initial geometric configuration when induced by a characteristic phase transformation related to two crystal structures, namely, martensite and austenite. The martensite phase is characterized by a monoclinic structure with twin planes. The crystal lattice allows great displacement thanks to a detwinning process originating from an applied load at the martensite finish transformation temperature (Mf), which does not induce bond breakage. The deformation takes place until it reaches a residual strain once the load has been removed. By heating the surface of the SMA component up to the austenite finish transformation temperature (Af), the phase transition from martensite to austenite is obtained, and the crystal lattice is arranged in an ordered orthorhombic structure. In this way, the SMA spring returns to its initial shape. The effect which is able to trigger the actuation is called the shape memory effect (SME). The transformation from austenite to martensite can be obtained by an applied load with a temperature higher than Af. This effect is known as stress induced martensite (SIM) effect and it induces stiffness variation from high values (characteristic of the austenite) to low values (characteristic of the martensite) [11,12,13]. Several numerical and analytical models able to describe SMA behavior can be found in the literature [14,15,16]. The Preisach hysteresis model has been chosen by many researchers and engineers as an excellent modeling approach to describe the behaviors of different shape memory alloys compositions [17,18,19]. In [20], a one-dimensional constitutive model of a SMA system was developed while taking into account the material characteristics involved with the internal phase transformation. The resistance characteristics of the SMA actuator during actuation were investigated in [21] as a function of the resistivity, length, and cross-sectional area. Experimental data were used to elucidate the mechanical and the electrical parameters of the specified SMA element.

In recent years, actuators based on shape memory alloy components have become lighter and smaller, and their use in all industrial fields has increased [22,23,24,25,26]. Nevertheless, especially in the aerospace field, experimental tests aimed at validating and certifying the mechanical behaviors of SMAs are mandatory. Rey et al. [27] studied the response of a SMA-based actuator for a variable area nozzle. The investigated device has been found to be two times lighter than a conventional mechanical system, significantly reducing the direct aircraft operating cost (~2–3%). Other applications and experimental results can be found elsewhere [28,29,30].

Lu et al. [31] developed a novel design for a parallel gripper actuated by a large-stroke shape memory alloy element. The use of multiple SMA wires characterized by connection in series has resulted in a larger actuated strokes in lightweight and smaller volumes for devices.

Indeed, all the previous cited works make us confident about the suitability of SMAs as actuators in engineering applications. Hence, it is mandatory to develop more and more sophisticated preliminary design tools able to effectively define the characteristics of SMA-based actuators according to design requirements.

In this work, a preliminary design procedure is proposed to conceptualize and properly size SMA actuators that has been developed and applied to a real prototype actuator. The procedure takes two different analytic models into account, namely, the An model [32] and the Spaggiari model [33], in order to build an overall engineering frame to recursively design a SMA actuator from an predefined shape memory spring. Indeed, exploiting the An model, it is possible to determine the mechanical properties of spring materials via simple tensile test data (micromechanics) which would be input to the Spaggiari model to size the entire actuator and determine the optimal functional point once the geometrical constraint of the physical dimensions of the actuator frame are fixed (macromechanics).

The procedure has been preliminary validated on a structure made of a SMA spring (CuNiTi) and two antagonist bias standard steel springs. The following constraints, taken from an adaptive aerodynamic automotive application, were assumed for designing the final SMA-based actuator prototype: (a) 30 N biased compression force; (b) 6 mm actuation; (c) a fixed actuator length of 68 mm; and (d) a final extra force of 85 N.

In Section 2, the theoretical background of the introduced analytical models is presented. Then, in Section 3, the results from the experimental tests used to evaluate the shape memory alloy properties necessary for the adopted macromechanical model are shown, while, in Section 4, the steps in designing the actuator, starting from the aforementioned design requirements, are introduced. Finally, in Section 5, the reliability of the proposed recursive procedure is assessed by comparing the data from the experimental tests on the designed actuator.

## 2. Theoretical Background

**Figure 1:**
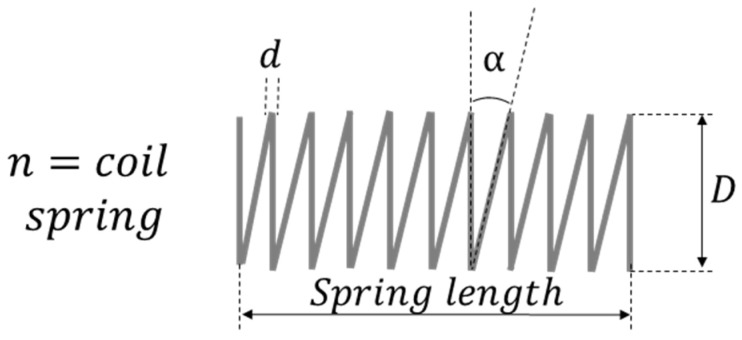
Geometric parameters of the tested springs.

The proposed preliminary design procedure uses analytical models to derive the mechanical behavior of a given bias and SMA springs for a SMA actuator and to size and design the actuator. In particular, the An analytical model [32] has been implemented for the evaluation of the mechanical properties of the spring components. In his work, An developed a novel design model for a single SMA coil spring actuator, starting from the formulae of the conventional coil spring design [32]. The mechanical behavior of the actuator is described in two distinct phases (100% martensite and 100% austenite) when following static modeling. Hence, deflection *δ*, force *F*, shear stress *τ*, and shear strain *γ* can be related to the geometric parameters of the spring according to Equations (1)–(4):

(1) $$\delta =\frac{\pi nD_{i}}{\cos\alpha_{i}}\left(\sin\alpha_{f}-\sin\alpha_{i}\right)$$

(2) $$F=\frac{\frac{\pi d^{4}G}{8D_{i}^{2}}\;\cos^{2}\alpha_{i}\left(\sin\alpha_{f}-\sin\alpha_{i}\right)}{\cos^{2}\alpha_{f}\left(\cos^{2}\alpha_{f}+\frac{\sin^{2}\alpha_{f}}{1+\upsilon }\right)}$$

(3) $$\tau =\frac{F(\frac{D}{2})(\frac{d}{2})}{\frac{\pi d^{4}}{32}}=\frac{8DF}{\pi d^{3}}=\frac{8CF}{\pi d^{2}}$$

(4) $$\gamma =\frac{1}{C}\frac{\cos^{2}\alpha_{i}\left(\sin\alpha_{f}-\sin\alpha_{i}\right)}{\cos^{2}\alpha_{f}\left(\cos^{2}\alpha_{f}+\frac{\sin^{2}\alpha_{f}}{1+\upsilon }\right)}=\frac{\tau }{G}$$

where, according to Figure 1 (describing the geometrical characteristics of the tested springs), *n* is the number of coils, *D* is the spring diameter, *α* is the pitch angle, *d* is the wire diameter, *G* is the shear modulus, *υ* is the Poisson’s ratio, and *C* is the spring index (*D*/*d*). Moreover, in Equations (1)–(4), the subscript “*i*” refers to the initial condition (deflection = 0), while the subscript “*f*” refers to the final condition (deflection = *δ*).

**Figure 2:**
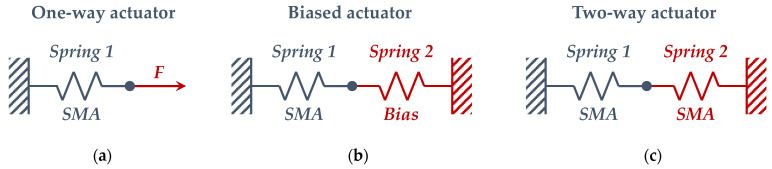
SMA-based actuator schemes with different antagonistic elements: (**a**) constant load; (**b**) antagonistic traditional spring; (**c**) antagonistic SMA spring.

By using experimental data (introduced in the next section) with the tested springs and Equations (1)–(4), the material properties of the tested bias and SMA springs have been derived. Then, in order to size and design the entire actuator, the Spaggiari model [33] has been used. Thanks to this model, in this paper, the characteristics of a SMA-based actuator, composed of SMA and antagonistic bias elements (see Figure 2) have been obtained starting from available SMA and bias springs. Hence, the device can be designed, according to geometrical and forces constraints, by choosing the appropriate number of coils for a given SMA spring. Of course, the Spaggiari model can be used in more general conditions where more than one geometrical parameter of SMA the spring is unknown.

In [33], for each actuator scheme described in Figure 2, a dissipative force and a conservative force have been considered. The first force can be associated to the friction, and it always acts in the opposite direction with respect to the velocity vector (the highest value between static and dynamic friction forces is considered). The second force is considered always acting on the actuator, such as gravity. As for the An model, the Spaggiari model considers a static modelling of the actuator as well, taking into account only the initial (martensite) and final (austenite) states. Hence, the four dimensionless coefficients introduced in Equations (5)–(8) can be defined, which depend on the material mechanical properties and on the forces carried out by the actuator:

(5) $$s_{1}=\frac{K_{SH}}{K_{SC}}=\frac{E_{A}}{E_{M}}=\frac{G_{A}}{G_{M}}$$

(6) $$s_{2}=\frac{K_{Bias\left(min\right)}}{K_{SC}}$$

(7) $$S_{F}=\frac{F_{F}}{K_{SC}\;\Delta x_{Max}}$$

(8) $$S_{0}=\frac{F_{0}}{K_{SC}\;\Delta x_{Max}}$$

where *K_SH_* and *K_SC_* are the stiffness of the SMA component in the hot and cold phases respectively, *E* is the elastic modulus, *K_Bias_*_(*min*)_ is the minimum value assumed by the stiffness of the bias spring, *F_F_* is the design dissipative force, *Δx_Max_* is the maximum deflection in the cold state, and *F*_0_ is the conservative force. Moreover, the subscripts “*A*” and “*M*” refer, respectively, to the austenite and martensite phases.

Through the coefficients introduced in Equations (5)–(8), the force balance can be imposed on both the cold (martensite) and hot (austenite) phases, aimed to derive the stiffness, the pre-compression (or pre-stretch), the maximum deflection, and the geometric characteristics of the actuator.

In this specific work, the actuator is described by scheme (**b**) in Figure 2. Due to the design constraints, it is possible to simplify the previous analytical procedure.

**Figure 3:**
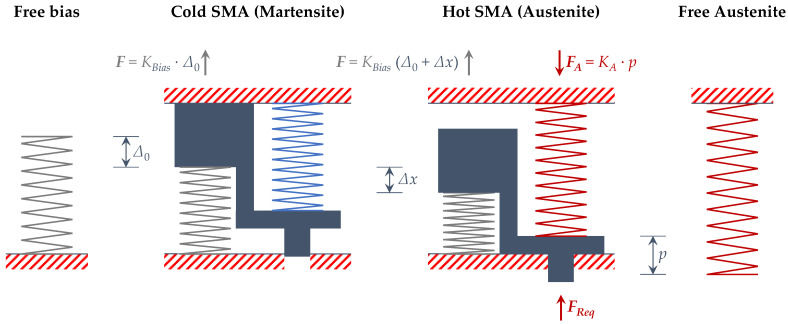
SMA-based actuator free body diagram.

A force balance has been performed on the hot phase of the SMA actuator in order to correlate the stiffness of the SMA spring in the austenite phase (*K_A_*) to the exerted force and stretching:

(9) $$K_{Bias}\;\left(\Delta_{0}+\Delta x\right)=K_{A}\;p-F_{Req}$$

where, according to the free body diagram shown in Figure 3, *K_Bias_* is the stiffness of the bias spring (obtained through the previously performed experimental tests), Δ_0_ is the compression of the bias spring with respect to its original shape considering the SMA spring in its cold (martensite) phase, Δ*x* is the stroke of the actuator, *p* is the compression of the SMA spring from its original shape to its hot (austenite) phase, and *F_req_* is the force required to be exerted by the actuator assuming the SMA component in hot phase (in this work the required force is a prerequisite of the actuator design and it is equal to 85 N). 

The number of active coils of the SMA spring can be obtained from the SMA Spring parameters by means of the following relationship:

(10) $$n=\frac{G_{A}d}{8K_{A}C^{3}}$$

Indeed, Equation (10) describes the relationship between the force and the displacement when assuming small deformation. Then, the preliminary length of the SMA spring *L_SMA_*_(1)_ can be evaluated as:

(11) $$L_{SMA\left(1\right)}=n\;\left(\pi \;D\tan\alpha +d\right)$$

In Equation (11), the pitch angle of SMA spring *α* is assumed to be 4°.

A second preliminary length, *L_SMA_*_(2)_, has been computed by using a congruence equation:

(12) $$L_{SMA\left(2\right)}=p+L_{Actuator}-\Delta_{t}$$

where *L_actuator_* is the length of the actuator and Δ*_t_* is a thickness value, assumed equal to 2 mm. Finally, a parameter “*S*” is introduced, which defines the difference between *L_SMA_*_(1)_ and *L_SMA_*_(2)_:

(13) $$S=L_{SMA\left(1\right)}-L_{SMA\left(2\right)}$$

**Figure 4:**
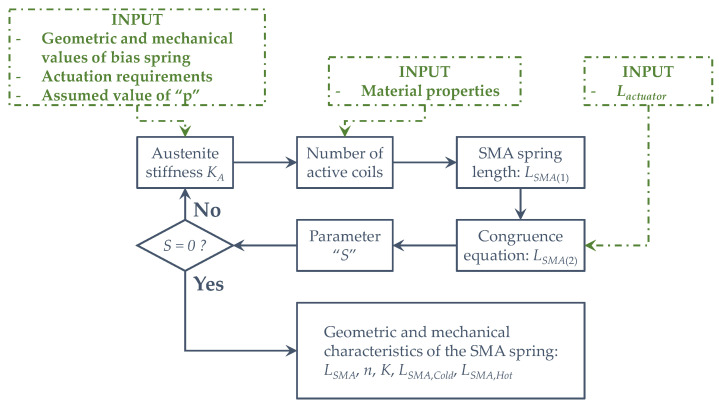
Design framework.

An Excel optimization code has been used, changing the parameter “*p*” until “*S*” reaches zero (Figure 4). Through these considerations, the preliminary design procedure of the actuator can be considered completed since all geometric and mechanical characteristics of the actuator have been obtained.

## 3. Experimental Setup

**Figure 5:**
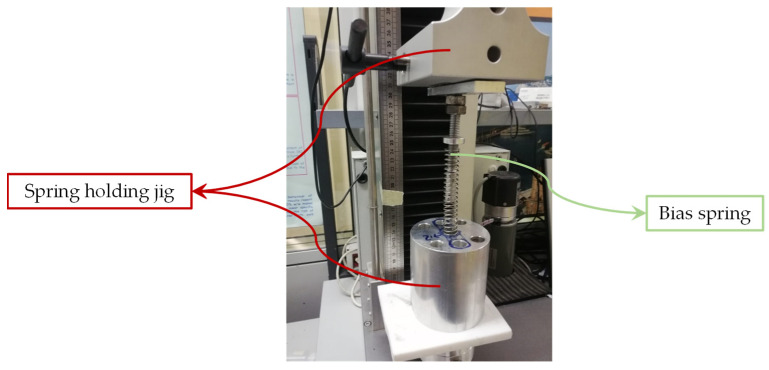
Bias spring during compression test.

The experimental phase has been conducted on two available components (steel bias spring and SMA spring). Several compression tests have been performed to obtain measurements of the stiffness, using an electro-mechanic UTM system (Lanos Test TT 2.5) with a load cell of 250 N. The steel spring was tested with a rate of 12 mm/min (Figure 5).

**Figure 6:**
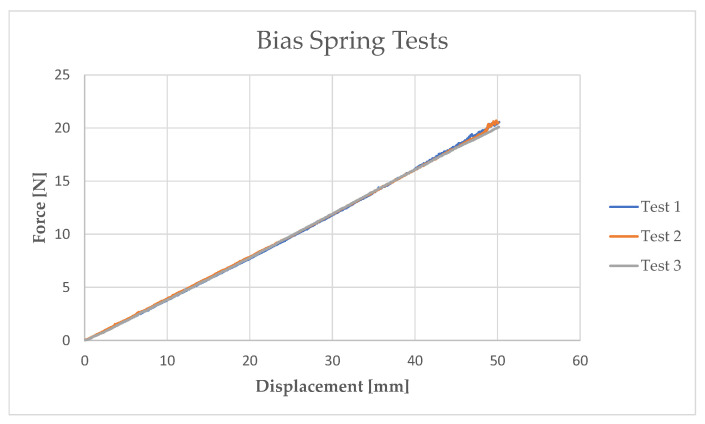
Bias (steel) spring compression tests.

**Figure 7:**
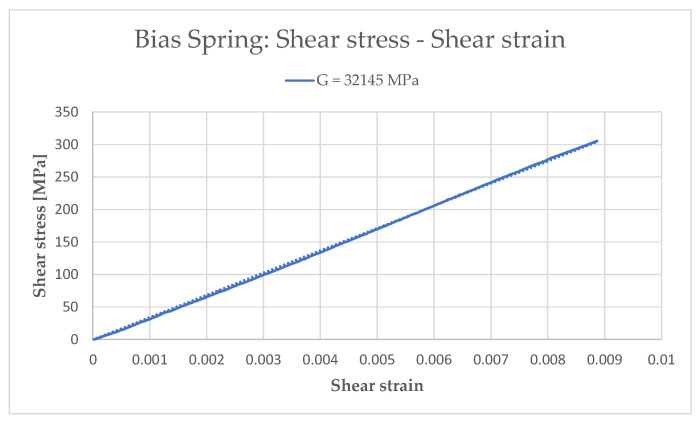
Shear stress–shear strain curve and corresponding shear modulus G.

Once the force and displacement data had been obtained (Figure 6), the corresponding stiffness value of the spring was derived. Then, the An model (Equations (1)–(4)) was used to determine the shear stress–shear strain curve and, consequently, shear modulus *G_Bias_* (Figure 7). The results for the bias spring are summarized in Table 1.

Subsequently, tests on the SMA spring have been performed considering different temperatures in order to fully describe the behavior of the shape memory alloy (NiTiCu).

Since shape memory alloy mechanical behavior strongly depends on the temperature, a differential scanning calorimetry (DSC) test has been preliminary performed. A SMA specimen with the weight of 6 mg was tested through a ramp of temperature with a rate of 10 °C/min.

**Figure 8:**
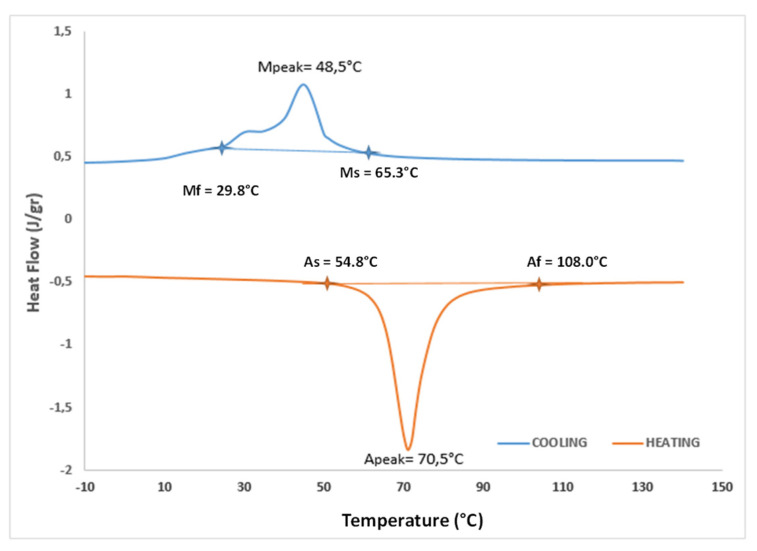
DSC test on the SMA specimen.

The heat flow monitored through temperature (Figure 8) allowed the definition of the characteristic temperature values reported in Table 2.

According to the transformation temperatures reported in Table 2, two different test temperatures have been defined. In particular, the spring in its martensite phase has been tested at temperature *T_ROOM_* equal to 25 °C, while the spring in its martensite phase has been tested at temperature *T_A_* equal to 100 °C.

**Figure 9:**
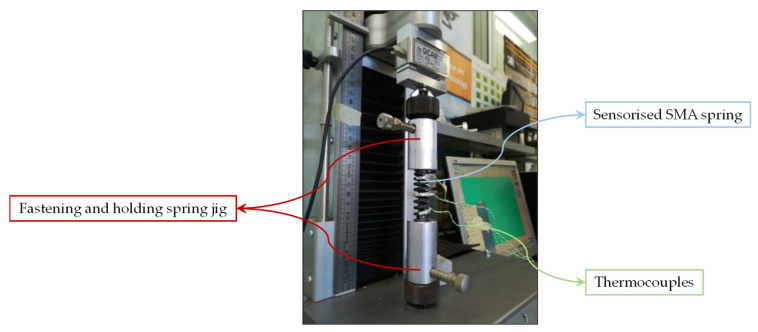
Sensorized SMA spring with thermocouples for the heated tensile test.

In order to define the complete behavior of the spring in its martensite phase, including also the detwinning process, several traction tests have been performed (Figure 9) on a martensite SMA spring whose geometrical characteristics have been taken into account in the analytical model. 

**Figure 10:**
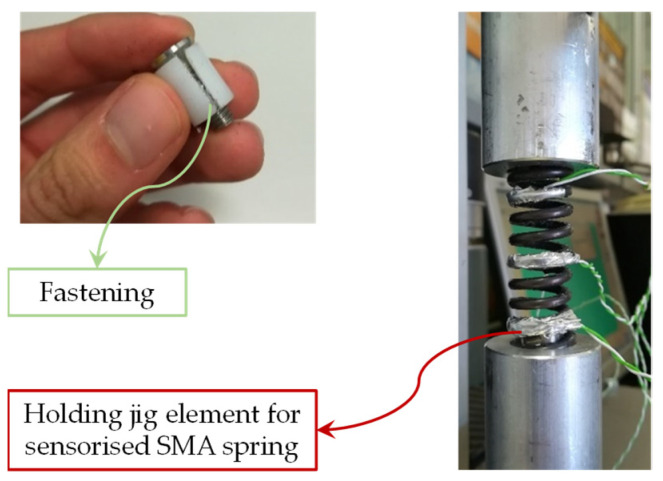
Fastening system for the SMA spring traction tests.

**Figure 11:**
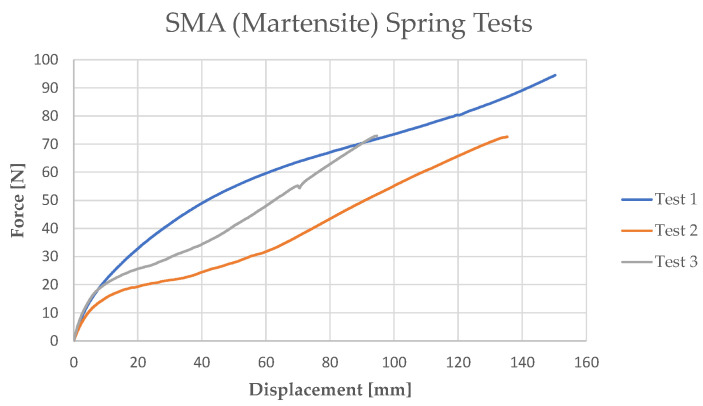
SMA (martensite) spring traction tests.

No traction hook was placed on SMA springs. Hence, to perform the tensile tests, it was necessary to design a locking system for the spring. A conical nylon cylinder was developed to be placed inside the spring. With the use of a screw, the diameter of the cone has been increased in order to produce friction on the internal surface of the spring. To avoid an increase in the spring diameter (*D*), a steel cylinder has been placed on the external surface of the spring in order to complete the fastening. Finally, the system has been connected to the load cell and the UTM (Figure 10). The results of the tensile tests are reported in Figure 11. Although the initial elastic region is likely repeatable, the curves reveal differences within at intermediate displacement due to unrecovered stretching. 

**Figure 12:**
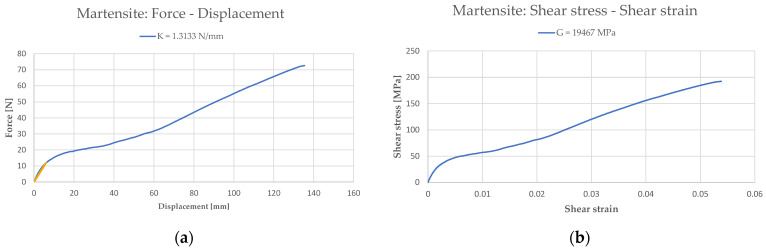
Mechanical and material properties of the martensite state: (**a**) force–displacement; (**b**) shear stress–shear strain.

Similar to what has been done for the bias spring, the An model (Equations (1)–(4)) has been used to derive the shear modulus *G_M_* from the normal force as a function of the axial displacement (Figure 12). The mechanical properties of the SMA spring in its martensite phase are summarized in Table 3.

**Figure 13:**
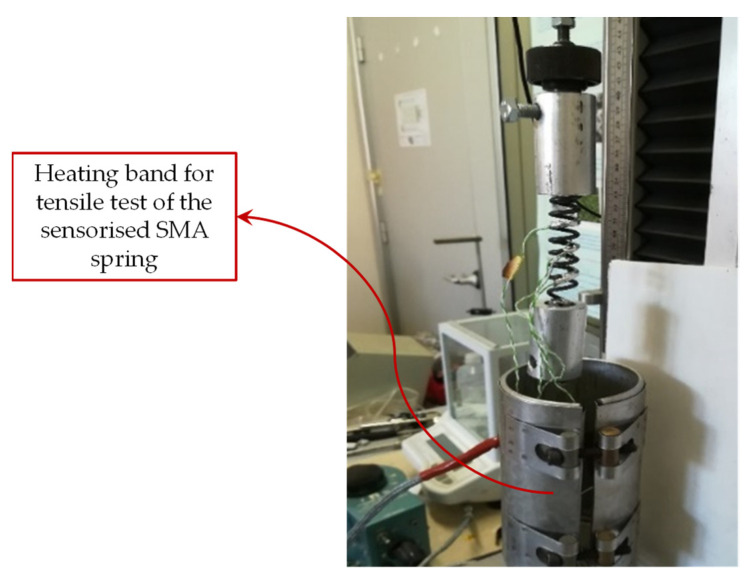
Electric resistance band used as a heating system.

**Figure 14:**
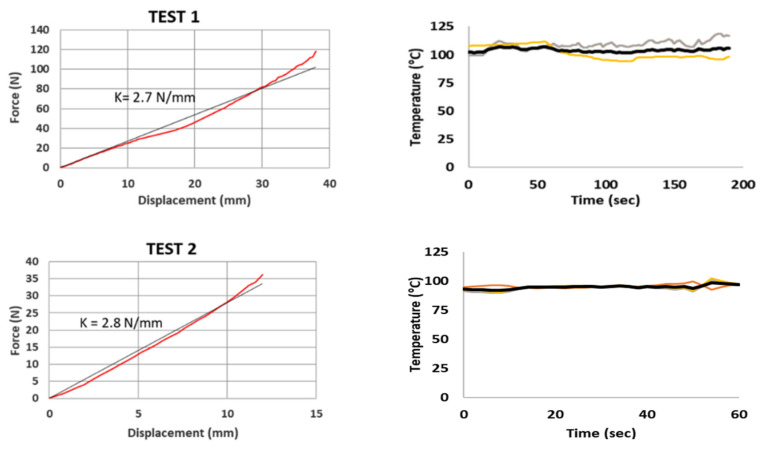

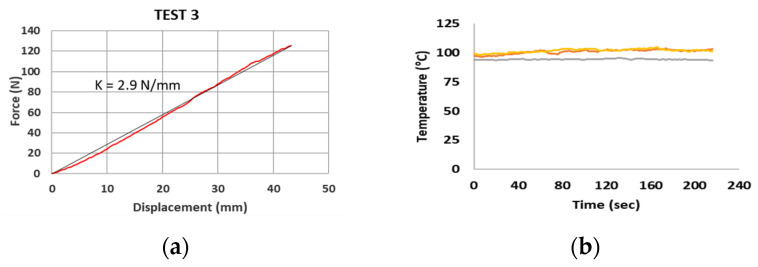
SMA (austenite) compression tests: (**a**) force–displacement; (**b**) temperature variation during time.

**Figure 15:**
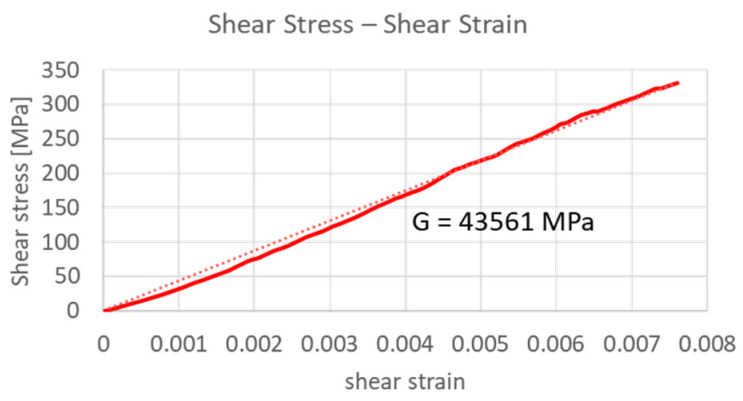
Material properties of austenite.

In order to test the SMA spring in its austenite phase, a heating system consisting of an electric resistance band has been used (Figure 13) to keep the surface temperature of the spring at a constant temperature of about 100 °C. The results of the tests are shown in Figure 14 and Figure 15 and summarized in Table 4.

The surface temperature has been monitored with three type “K” thermocouples. A number of compression tests have been executed with an austenite SMA spring whose geometrical characteristics have been taken into account in the analytical model.

## 4. Application of the Procedure to the Design of the Test Case Actuator

**Figure 16:**
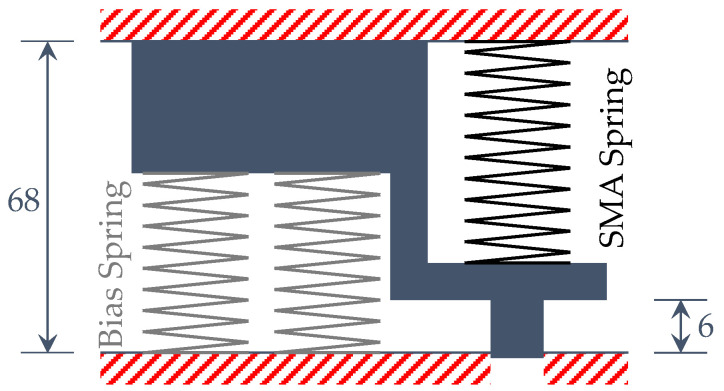
Geometrical arrangement and description of the investigated actuator (dimensions in mm).

The analytical procedure implemented in this work has been used, together with the experimental data introduced in the previous section, to design a SMA actuator based on available bias and SMA springs and is based on specific design requirements derived for an application to adaptive aerodynamics in automotive. The actuator has been considered to be composed of a single SMA spring (whose number of coils has to be determined) and two bias springs, as shown in Figure 16. The length of the actuator is a prerequisite and was considered fixed here. The springs have been placed in the actuator in a compressed position with respect to their original shape.

The two bias springs have been considered as a single equivalent steel spring, which must exert a pre-compression force on the SMA component equal to 30 N (according to design requirements). 

**Figure 17:**
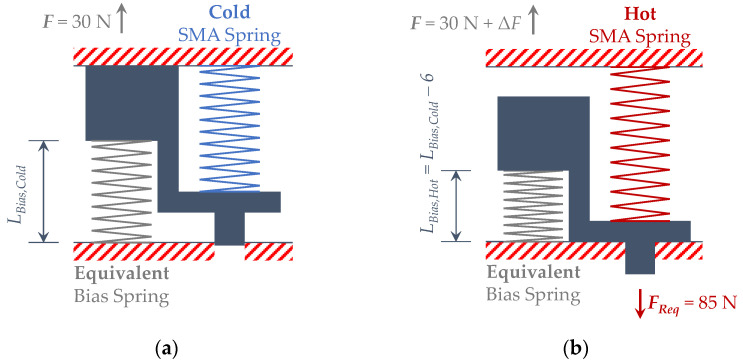
Operating scheme of the actuator (dimensions in mm). (**a**) Cold phase; (**b**) hot phase.

The actuator has been drafted (including the fixed design requirements) in its two distinct martensite 100% and austenite 100% phases, as reported in Figure 17.

The design procedure has been carried out within the analytical framework described in the previous sections. The mechanical characteristics of the bias spring ($K_{Bias_{equivalent}}=2\;K_{Bias}=0.8$), obtained from experimental tests, have been used as an input in Equation (9) while assuming the requirement force of 85 N. The compression produced by pre-compression force of 30 N led to:

- $\Delta_{0}=\frac{30}{0.8}=37.5\;\mathrm{mm}$
- $\Delta x=6\;\mathrm{mm}\;\left(\mathrm{actuator}\;\mathrm{stroke}\right)$

A trivial initial value has been assigned to the “p” parameter, so that the analytic procedure can be initialized and the austenite stiffness $K_{A}$ has been obtained. Considering the material properties ($G_{A}$), Equation (10) was used to determine the number of active coils (n) of the SMA spring by assuming the following values for the SMA spring and for the wire:

- d=2.5 mm
- D=16.25 mm
- $C=\frac{D}{d}=6.5$

Moreover, with the assumption of $\alpha_{SMA}=4{}^{\circ}$, the length of the spring was determined by using Equation (11). The parameter $L_{SMA\left(2\right)}$ is calculated by means of the congruence Equation (12) and, the parameter “*S*” was initialized.

The results of the analytical process, iterated until the parameter “*S*” is equal to zero as described in Section 2, are summarized in Table 5 and Table 6. The “Cold” and “Hot” subscripts describe the two different configurations of the SMA spring shown in Figure 17.

## 5. Results and Experimental Validation on the SMA Actuator

On the basis of the results obtained from the analytical procedure, a SMA actuator consisting of one SMA spring, whose geometrical and mechanical properties are reported in Table 5, and two bias springs (Table 6) has been designed. In this section, the testing of designed actuator is introduced. In Table 7, the geometric characteristics of the bias and the SMA spring derived from the analytical procedure and the one actually manufactured (due to specific manufacturing constraints) and tested are compared.

**Figure 18:**
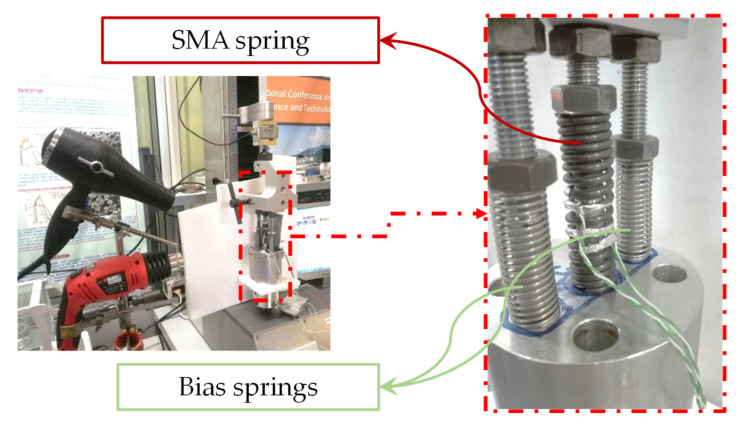
Heating and cooling system for actuator test and zoomed image of the sized bias-SMA based springs.

Static tests have been carried out by using the UTM (Lenos Test) with a 250 N load cell to detect the maximum force exerted by the SMA spring. A locking system has been used to keep in position the actuator, in order to perform the tests in the hot and cold conditions. To achieve the austenite 100% state, a trivial heating system able to keep the temperature in the range 90 °C–110 °C has been used. Actually, two tests (TEST 1 and TEST 2) have been carried out, respectively at 90 °C and 110 °C. Moreover, forced cooling and heating cycles (Figure 18) have been carried out in order to detect the force peaks of the SMA spring when temperature reaches the minimum value of about 40 °C.

**Figure 19:**
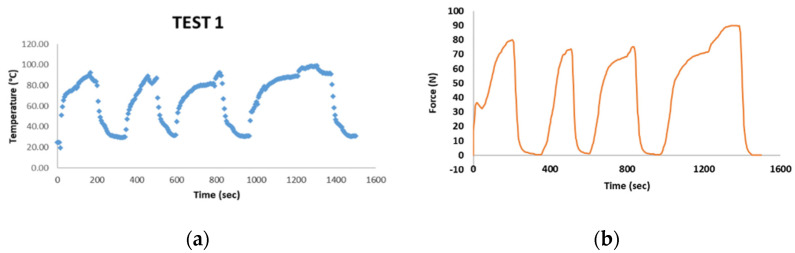
Experimental test results with a heat peak achieved of 90 °C: (**a**) temperature–time, (**b**) force–time.

The results of test 1, shown in Figure 19, highlight that the maximum temperature peak achieved of 90 ± 5 °C corresponds to a force exerted by the SMA spring of about 80 ± 5 N. This is representative that for this temperature, the martensite to austenite transformation has been not extended to the entire SMA spring volume. 

**Figure 20:**
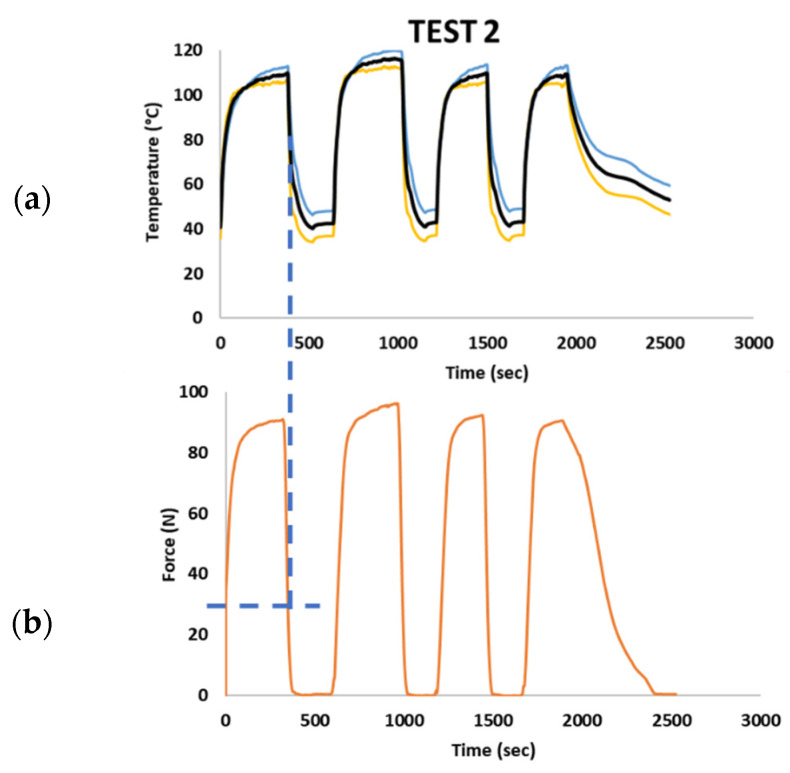
Experimental test results with a heat peak achieved of 110 °C: (**a**) temperature–time; (**b**) force–time.

Test 2 (Figure 20) was carried out with a temperature peak of 110 ± 5 °C. The increase in temperature led to a higher austenite volumetric coefficient. Hence, experimental tests returned, as a result, a maximum load of 90 ± 5 N, which is very close to the designed loading conditions. These experimental results highlight an issue in the design procedure. As known, martensite to austenite transformation is a gradual process taking place within a significant temperature range. This consideration suggests taking into account the volumetric fraction of the transformed austenite in the design process in order to provide a more realistic mechanical prediction representative of the real loads exerted at operative temperatures.

## 6. Conclusions

In this work, a preliminary design procedure for the definition and sizing of SMA actuators has been introduced. Two analytical static models have been identified and integrated in the preliminary design procedure. Indeed, by using the An model, the material properties of SMA and bias springs can be derived. Then, the Spaggiari model has been used to design the actuator, composed of an antagonist SMA and bias springs while taking into account design constraints and providing the definition of one or more geometrical parameters of the SMA spring. These analytical models, combined in the proposed procedure, have been used to design a SMA actuator with specific design requirements that is capable of exerting a maximum force of 85 N. In order to design and validate the developed procedure, a number of experimental tests have been performed. In particular, tests on the spring elements (bias and SMA) have been performed to obtain the material properties needed for the application of the proposed design procedure. Finally, additional tests have been carried out on the entire actuator system in order to confirm the actuation specifications and preliminary validate the proposed procedure. 

This experimental study has demonstrated that the investigated SMA actuator, designed on the basis of the geometric characteristics resulting from the proposed procedure, is able to exerts the desired force by heating the surface of the spring up to a temperature equal to 90 °C. Upon cooling, 6 mm of actuation can take place due to a preload on the bias springs as required. The application of the proposed design procedure can be of relevance to increase the use of shape memory alloys for actuator applications allowing to design lighter actuator systems if compared to the traditional hydraulic and/or electromechanical actuators.

## Figures and Tables

**Table 1:** Mechanical and material properties of the bias spring.

Property	Value	Unit
*K_Bias_*	0.4	N/mm
*G_Bias_*	32.14	GPa
*E_Bias_*	85.5	GPa

**Table 2:** Transformation temperature of the SMA specimen.

Phase Transformation	Transformation Temperature	Peak Temperature
From martensite to austenite (heating)	A_s_ = 54.8 °C A_f_ = 108.0 °C	70.5 °C
From austenite to martensite (cooling)	M_s_ = 65.3 °C M_f_ = 29.8 °C	48.5 °C

**Table 3:** Specific material values for martensite.

Property	Value	Unit
K_M_	1.3	N/mm
G_M_	19.5	GPa
E_M_	51.6	GPa
$\tau_{Ms}^{cr}$	35	MPa
$\tau_{Mf}^{cr}$	79	MPa
*γ_L_*	0.0128	-
*ν*	0.33	-

**Table 4:** Specific material values for austenite.

Property	Value	Unit
K_A_	2.8	N/mm
G_A_	43.6	GPa
E_A_	115.9	GPa
$\tau_{A}^{cr}$	330	MPa
ν	0.33	-

**Table 5:** Simulated results for the SMA spring.

Property	Value	Unit
p	40.36	mm
G_A_	2.83	N/mm
n	18	-
L_SMA_	106.2	mm
L_SMA,Hot_	64	mm
L_SMA,Cold_ = L_SMA,Hot_ − Δx	58	mm

**Table 6:** Mechanical and geometrical values assumed for the design of the bias spring.

Property	Value	Unit
K_Bias,equivalent_	0.8	N/mm
L_Bias_	85	mm
L_Bias,Hot_	41.5	mm
L_Bias,Cold_	47.5	mm

**Table 7:** Comparison between the geometric characteristics of bias and SMA spring obtained from the analytical procedure and experimentally tested.

Property	Analytical Procedure	Experimental Test	Unit
n_SMA_	18	18	-
L_SMA_	106.2	104 ± 1	mm
L_SMA,Hot_	64	65 ± 1	mm
L_SMA,Cold_	58	59 ± 1	mm
L_Bias,Hot_	41.5	41 ± 1	mm
L_Bias,Cold_	47.5	47 ± 1	mm
